# Changes in body weight and food choice in those attempting smoking cessation: a cluster randomised controlled trial

**DOI:** 10.1186/1471-2458-12-389

**Published:** 2012-05-29

**Authors:** Wilma S Leslie, Preethi R Koshy, Mhairi Mackenzie, Heather M Murray, Susan Boyle, Michael EJ Lean, Andrew Walker, Catherine R Hankey

**Affiliations:** 1 Human Nutrition, University of Glasgow, 4th Floor Walton Building, Glasgow Royal Infirmary, Castle Street, Glasgow, G4 0SF, UK; 2 Urban Studies, University of Glasgow, Glasgow, UK; 3 Robertson Centre, University of Glasgow, Glasgow, UK; 4 Glasgow & Clyde Weight Management Service, Mansionhouse Unit, Mansionhouse Road, Glasgow, UK

## Abstract

**Background:**

Fear of weight gain is a barrier to smoking cessation and significant cause of relapse for many people. The provision of nutritional advice as part of a smoking cessation programme may assist some in smoking cessation and perhaps limit weight gain. The aim of this study was to determine the effect of a structured programme of dietary advice on weight change and food choice, in adults attempting smoking cessation.

**Methods:**

Cluster randomised controlled design. Classes randomised to intervention commenced a 24-week intervention, focussed on improving food choice and minimising weight gain. Classes randomised to control received “usual care”.

**Results:**

Twenty-seven classes in Greater Glasgow were randomised between January and August 2008. Analysis, including those who continued to smoke, showed that actual weight gain and percentage weight gain was similar in both groups. Examination of data for those successful at giving up smoking showed greater mean weight gain in intervention subjects (3.9 (SD 3.1) vs. 2.7 (SD 3.7) kg). Between group differences were not significant (p = 0.23, 95% CI −0.9 to 3.5). In comparison to baseline improved consumption of fruit and vegetables and breakfast cereal were reported in the intervention group. A higher percentage of control participants continued smoking (74% vs. 66%).

**Conclusions:**

The intervention was not successful at minimising weight gain in comparison to control but was successful in facilitating some sustained improvements in the dietary habits of intervention participants. Improved quit rates in the intervention group suggest that continued contact with advisors may have reduced anxieties regarding weight gain and encouraged cessation despite weight gain. Research should continue in this area as evidence suggests that the negative effects of obesity could outweigh the health benefits achieved through reductions in smoking prevalence.

**Trial registration:**

Current Controlled Trials ISRCTN73824458

## Background

Good nutrition is vital to good health and while many people eat well, a large number make poor food choices leading to poorer health. This is particularly evident in the more disadvantaged and vulnerable in the population [1]. Smokers are a well recognised vulnerable group, known to make poor food choices and consume an energy dense diet that is inferior in nutritional quality to that of non-smoking adults [2,3].

In the UK as in other developed countries obesity, with its many well-established negative implications for health, continues to increase in prevalence [4,5]. Weight change occurs over time and against a background of progressive age-related weight gain in the “normal” population. However, there are particular periods of life that are associated with weight gain and one such stage is smoking cessation [6]. Stopping smoking influences energy balance by reducing metabolic rate and by improving taste and appetite. Snacking is a common displacement activity after smoking cessation. Continuation of some of the poor food choices established prior to smoking cessation, and known to favour high-energy intakes, may exacerbate energy imbalance and encourage weight gain.

Around 80% of smokers gain weight following smoking cessation [7]. The magnitude of weight gain is variable but an average weight gain of 5-6 kg within 2 years is common [8-10]. Weight gain is thought to be greatest in the immediate post cessation period [8]. With over 25% of smokers already obese and at particular risk of gaining more weight following cessation, the problem of post-cessation weight gain can have huge health consequences. Smoking and obesity are both risk factors for cardiovascular disease and some cancers [7]. The additional weight gain experienced post cessation may be sufficient to make co-morbidities clinically apparent.

Fear of weight gain is a barrier to smoking cessation and significant cause of relapse for many [8,11]. Allaying anxieties regarding weight gain could lead to greater uptake of services, improvements in quit rates and reductions in the prevalence of obesity. A recent review has highlighted the difficulties of reducing post cessation weight gain using behavioural interventions [11]. Advice to control weight by calorie reduction may compromise abstinence and is not advised while other approaches including very low calorie diets and cognitive behavioural therapy may reduce weight gain without compromising abstinence. However caution is advised on use of the these approaches. Overall the data were considered insufficient to make strong clinical recommendations for effective programmes to prevent post cessation weight gain. It is therefore pertinent that investigation of approaches to minimise post cessation weight gain continues.

In Glasgow, Smokefree Services a community-based group behavioural counselling service based on the Maudsley Model [12] is available for those wishing to give up smoking. Although diet and weight issues are routinely raised as concerns by those attending these groups, nutrition is not formally addressed. Provision of dietary advice may assist some in smoking cessation and thus improve health from this point of view but also improve food choice and minimise weight gain. Improved diet composition could lead to better health even in those who fail to stop smoking. The aims of this study were to determine the effect of a structured programme of dietary advice based on healthy eating principles, on food choice and weight change, in those attempting smoking cessation.

## Methods

### Study design

**Figure 1:**
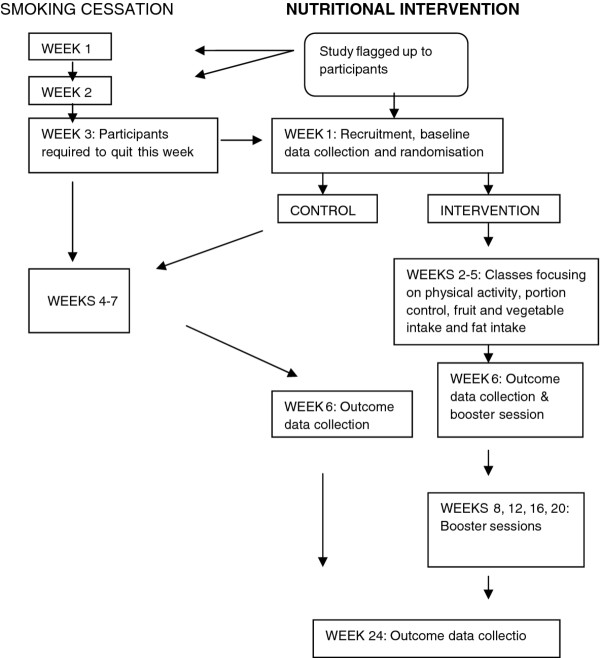
Study profile.

A cluster randomised controlled design was used with smoking cessation classes the unit of randomisation. Randomisation was carried out using an Interactive Voice Response system. Classes randomised to intervention commenced a 24-week intervention focussed on improving food choice and minimising weight gain. Classes randomised to control received “usual care” (Figure 1). Usual care comprised of 7 weeks of group support sessions. At the time of this study nutrition/diet was not formally addressed. Advice on physical activity was provided in one session and the availability of local leisure facilities to encourage improvements in physical activity promoted.

### Intervention

The intervention was delivered by smoking cessation advisors, who had undergone a 3-day nutrition and behavioural change training programme designed specifically for the study. The intervention comprised an initial 5-week programme of nutritional advice with a different aspect of nutrition covered at each add-on group session.

· Session 1) Energy balance and Physical activity

· Principless of energy balance and how it is affected

· Effects of smoking on energy balance

· Benefits of physical activity and effect on energy balance

· Session 2) Healthy Eating and Portion Control

· Healthy eating quiz

· Composition of healthy balanced diet, including portion size

· Session 3) Fruit and Vegetables

· Five-a-day target

· Portion sizes for fruit & vegetables

· How to achieve 5-a-day

· Session 4) Reducing Fat Intake

· Different types of fat

· Contribution of fats to weight gain

· Food labelling and identification of foods high in saturated fats

· Session 5) How to Keep it all going

· Review of previous topics and participants’ progress

This was followed by 5 additional follow up group sessions for review and reinforcement of advice, and self monitoring.

All sessions comprised of advice/information, group discussion, sharing of experiences and peer support and mirrored the format of smoking cessation sessions. Healthy Living Packs produced by the Scottish Executive (http://www.healthscotland.com), were given to provide volunteers with written information. Packs contained recipe cards, and information on the following topics: - labelling claims and labels, sugars, salts and fats, eating for health and a healthy eating quiz, and food safety. Plate models, a tool to assist dietary education, were provided to direct the composition of main meals [13].

The intervention did not include a formal structured physical activity element but the benefits of physical activity were discussed and increases encouraged. Physical Activity packs and pedometers (SW-200 Digi-Walker, Yamax Corp) were provided to encourage improvements.

A manual, detailing the content of each session, the measurements to be taken and the tools/leaflets to be distributed was produced and used by all advisors.

### Measurements

Intervention and control participants had measurements of body weight, waist circumference and food choice/intake made at baseline, weeks 6 and 24. Dietary practices were assessed using the Dietary Targets Monitor [14]. Measurements of exhaled carbon monoxide (CO) were made at weeks 6 and 24 to assess smoking status (piCO + breath CO monitors, Bedfont Scientific Ltd).

Measurements were recorded by the trained smoking cessation advisors. A member of the research team attended all week 6 and 24 follow-up sessions to maximise completeness of data collection.

A small financial incentive (£10 retail voucher) was offered to all participants to encourage attendance at week 6 and week 24 follow-up sessions to have repeat measurements taken.

### Sample size

The study sought to minimise the 5% weight gained usually associated with smoking cessation. It was considered that a difference between the weight change of the control and intervention groups of ≤3% would be of clinical importance, and sufficient to judge the success of the intervention [15]. Due to no prior knowledge of an estimate for the intra-cluster correlation (ICC) a conservative range of 0.05 to 0.15 was assumed. Assuming a significance level of 0.05, the study would have 78, 83, 88% power for ICCs of 0.15, 0.1 and 0.5 assuming 5 completers per class. To allow for a potential attrition rate of 75% it was planned to recruit 16 classes with an average of 10 participants for each treatment arm (total of 32 classes). A minimum of 40 completers per arm and class sizes of 5 or more were required to fulfil the sample size requirements.

### Outcome measures

The primary outcome was difference in % weight change at 24 weeks from baseline in the diet intervention group compared to control group. Secondary and tertiary outcomes were change in body weight at 24 weeks from baseline, and measures of food choice. The study was not powered to detect changes in smoking status.

### Statistical analysis

Random effect models adjusted for baseline values and varenicline (Champix, Pfizer Ltd) use were used for analysis of the primary, secondary and tertiary outcome measures to account for clustering of smoking cessation centres. Intracluster correlation coefficients (ICC) were reported along with adjusted mean differences (95% confidence interval (CI) and corresponding p-values.

Logistic regression models adjusting for cluster (i.e. smoking cessation classes) were used to analyse binary outcomes (such as smoking status, and favourable changes in food intake at 24 weeks from baseline). A sensitivity analysis was further performed for smoking status assuming the ‘worst’ case scenario that all participants dropping out of the study had resumed smoking. General linear models and ordinary logistic regressions models ignoring clustering were also used to analyse continuous and categorical outcomes respectively, as the ICC for many of the outcomes were small or less than zero (outcomes with negative ICC were truncated at zero for the analysis).

Changes in the intake of key foods groups at 24 weeks from baseline were compared separately for the two study groups using a Sign Test. Food intake was summarised as the number of participants who had either decreased, remained the same or increased their food intake at 24 weeks from baseline.

### Process evaluation

A process evaluation of the intervention was carried out to understand the context in which specific mechanisms that influenced weight management and smoking cessation were triggered among participants. Intervention and control participants were interviewed at weeks 6 and 24. At week 6, a purposive sample of 15 pairs of participants was matched for BMI at recruitment and cigarette consumption. To account for drop-outs at 6 months, 15 pairs of participants were sampled after matching for BMI at recruitment. Interviews were semi-structured, audio-taped with participants’ consent, transcribed verbatim and entered into ATLAS-ti software (ATLAS/Ti for Windows 1996). Participants were interviewed at the class venue and if this was not possible, a telephone interview was carried out. All interview transcripts were anonymised and given an identification number. The transcripts were coded, thematically analysed according to *a priori* and emergent themes and then the themes were linked to develop explanations. During this process, the text was purposively searched for contradictory data and emerging themes and explanations were discussed and agreed by the research team. Process evaluation results will be fully reported in a separate paper and are summarised only in the present paper.

### Economic evaluation

An economic evaluation was carried out to determine the costs of delivering the intervention, compared to the benefits. The perspective taken was that of the NHS for costs (and savings), and health benefits to patients measured in Quality-Adjusted Life-Years (QALYS), a generic outcome measure commonly used in health technology assessment which capture changes in ‘quality of life’ (health status) and length of life, in a way that is comparable across many different therapy areas. This reflects the approach used by the National Institute for Health and Clinical Excellence (NICE) for appraising new technologies [16]. Costs of the intervention included staff time, room hire, plus costs of handouts and other printed material. An exploratory analysis extrapolating the changes in weight into QALYs was proposed by extrapolating from the trial data using previously published methods [6,17].

Ethical approval was secured on 18^th^ September 2007, from the West Ethics Committee Glasgow.

## Results

### Recruitment

**Figure 2:**
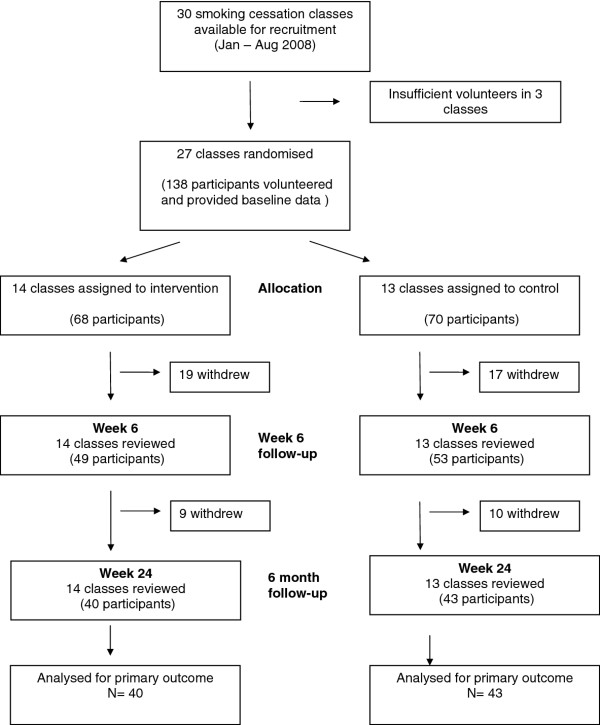
Flow chart showing recruitment to the study.

Recruitment was carried out between 1^st^ January and 31^st^ July 2008. Thirty smoking cessation classes were available for recruitment, 3 could not be randomised as only one person volunteered to participate. Twenty-seven classes (138 participants) were randomised and entered the study (Figure 2). The median number of participants per class was 5 (range 2-13).

### Baseline measurements

Baseline demographic, anthropometric and smoking habit characteristics of participants in the control and intervention arms were similar (Table 1). The majority were female (75.4%), mean age 50 years. The reported dietary habits of both groups were broadly similar fruit and vegetable consumption was low and foods high in fat or sugar were eaten regularly (Table 2).

**Table 1:** Baseline characteristics of all participants recruited to the study

	**Intervention (n = 68)**	**Control (n = 70)**	**Total (n = 138)**
**Anthropometric**	Mean (SD)	Mean (SD)	Mean (SD)
Weight (kg)	76.3 (16.9)	76.1 (19.2)	76.2 (18.0)
BMI (kg/m^2^)	28.4 (5.4)	28.0 (5.7)	28.2 (5.5)
Waist circumference (cm)	95.0 (13.2)	92.3 (14.9)	93.7 (14.1)
**Smoking Data**			
Time smoked (Years)	32.0 (14.0)	30.3 (11.5)	31.1 (12.8)
Cigarettes/day	26.0 (14.1)	24.4 (10.9)	25.2 (12.6)
Previous quit attempts	2.6 (1.8)	3.1 (2.1)	2.8 (2.0)
**Product used for current quit attempt**	**n (%)**	n* (%)	**n (%)**
Varenicline (Champix)	35 (51.5)	46 (66.7)	81 (59.1)
Patch/Patch and gum	20 (29.4)	13 (18.8)	33 (24.1)
Gum	4 (5.9)	2 (2.9)	6 (4.4)
Lozenge	0 (0.00)	2 (2.9)	2 (1.5)
Inhalator	6 (8.8)	3 (4.4)	9 (6.6)
Microtab	0 (0.00)	1 (1.4)	1 (0.73)
Nasal spray	3 (4.4)	2 (2.9)	5 (3.6)

* product unknown for one control subject.

**Table 2:** **Baseline Self-reported Eating Habits** (Data presented as median and inter-quartile range)

	**Intervention (n = 69)**	**Control (n = 69)**		**Intervention (n = 69)**	**Control (n = 69)**
**All fruit and veg (day)**	3.0 (2–5)	2.1 (1–4)	**Red meat (wk)**	3.0 (3–3)	3.0 (1–3)
**Slices bread/rolls (day)**	2.5 (3–5)	2.5 (1–3)	**Meat products (wk)**	3.0 (1–3)	3.0 (1–3)
**Breakfast cereal (wk)**	3.0 (0–7)	3.0 (0–7)	**Poultry (wk)**	3.0 (1–3)	3.0 (1–3)
**Potatoes/rice/pasta (wk)**	5.5 (3–7)	3.0 (3–7)	**White fish (wk)**	1.0 (0–1)	0.5 (1–1)
**Beans/pulses (wk)**	1.0 (1–3)	1.0 (1–3)	**Oil rich fish (wk)**	0 (0–1)	0 (0–1)
**Chips (wk)**	1.0 (1–3)	1.0 (1–3)	**Biscuits (wk)**	3.0 (1–18)	3.0 (1–7)
**Cheese (wk)**	3.0 (1–3)	3.0 (1–6)	**Cakes/pastries (wk)**	1.0 (0–3)	1.0 (1–6)
**Crisps/savoury snacks (wk)**	1.0 (1–3)	3.0 (1–7)	**Soft/fizzy drinks (wk)**	2.5 (0–7)	3.0 (0–18)
**Sweets/chocolates (wk)**	3.0 (1–7)	3.0 (1–7)			

### Attrition

At week 24 the overall attrition rate was 40%, with similar numbers seen for both groups (intervention group - 28 (41%) and control group – 27 (39%). In both groups the majority (67%) who withdrew dropped out of the entire smoking cessation programme rather than just this study so no further information could be obtained. Eighty-three participants (40 intervention 43, control) completed the 24 week study period (Figure 2). Baseline characteristics were similar for completers as for all participants (Table 3).

**Table 3:** Baseline characteristics of participants who completed the study

	**Intervention (n = 40**	**Control (n = 43)**	**Total (n = 83)**
**Anthropometric**	Mean (SD)	Mean (SD)	Mean (SD)
Weight (kg)	76.9 (18.5)	74.4 (18.0)	75.6 (18.2)
BMI (kg/m^2^)	28.7 (5.8)	27.7 (4.8)	28.2 (5.3)
Waist circumference (cm)	95.3 (13.9)	92.1 (13.1)	93.6 (13.5)
**Smoking Data**			
Time smoked (Years)	34.1 (14.8)	34.4 (10.8)	34.3 (12.8)
Cigarettes/day	25.0 (10.2)	25.3 (12.5)	25.2 (11.4)
Previous quit attempts	2.7 (1.9)	2.6 (1.7)	2.6 (1.8)
**Product used for current quit attempt**	**n (%)**	**n (%)**	**n (%)**
Varenicline (Champix)	21 (52.5)	26 (60.4)	47 (56.6)
Patch/Patch and gum	13 (32.5)	9 (20.9)	22 (26.5)
Gum	2 (5.0)	2 (4.6)	4 (4.8)
Lozenge	0 (0.00)	1 (2.3)	1 (1.2)
Inhalator	3 (7.5)	2 (4.6)	5 (6.0)
Microtab	0 (0.00)	1 (2.3)	1 (1.2)
Nasal spray	1 (2.5)	2 (4.6)	3 (3.6)

### Weight Change: (0-24 weeks)

Similar increases in weight were seen in the intervention and control group (Table 4). In comparison to the intervention group more control group participants minimised weight gain to ≤ 3% body weight (Table 4). However no statistically significant between group differences were seen in percentage weight gain, body weight, waist circumference and BMI (Table 5). These data however include those who continued to smoke.

**Table 4:** Changes in weight and waist circumference between baseline and week 24

**Variable**	**Intervention (n = 40)**	**Control (n = 43)**
**% weight change**	3.6 (4.7)*	2.8 (4.6)
**Weight (kg)**	2.6 (3.4)	2.0 (3.5)
**Waist circumference (cm)**	0.5 (3.8)	2.0 (4.4)
**% subjects gaining ≤3% body weight**	18 (45.0)**	25 (58.1)
**% subjects gaining > 3% body weight**	22 (55.0)	18 (41.9)

* mean (SD).

** n (%).

**Table 5:** Comparison of changes in outcome measures between intervention and control group at week 24

**Outcome**	**ICC**	**Adjusted Mean Difference (Intervention-Control)**	**Adjusted 95% C.I. of Difference**	**p**
**Primary outcome**				
Percentage weight change	0.015	0.904	−1.206 to 3.013	0.39
**Secondary outcomes**				
Change in weight (kg)	0.025	0.540	−1.060 to 2.139	0.50
Change in waist circumference (cm)	0.239	−1.170	−3.494 to 1.155	0.32
Change in BMI (kg/m^2^)	0.000	0.158	−0.437 to 0.753	0.60

analyses are random effects linear regression models adjusted for baseline values and clustering by centre. Negative ICC are truncated at 0.

Analysis of weight change in those successful at stopping smoking was underpowered (66%) but indicated greater mean weight gain in intervention subjects (3.9 (SD 3.1) vs. 2.7 (SD 3.7) kg). Between group differences were not significant (p = 0.23, 95% CI −0.9 to 3.5) however the confidence interval is wide and includes a loss of −0.9 kg, thus the possibility of an effect in favour of the intervention cannot be excluded.

In those not smoking a similar proportion of intervention and control subjects minimised weight gain to ≤ 3% (34.8% vs. 33.3%; p = 0.94, 95% CI 0.26 to 4.25).

### Eating Habits: (0-24 weeks)

In comparison to baseline some statistically significant improvements were observed in the self-reported dietary habits of the intervention group. Fruit and vegetable consumption increased significantly as did breakfast cereal intake. These changes were not seen in the control group (Table 6). However no between group differences were seen in the proportion of participants favourably changing their eating habits (Table 7).

**Table 6:** Within group changes in Eating Habits between baseline and week 24 for Intervention (n = 40) and Control groups (n = 43)

**Food Group**	**Group**	**Decrease**	**Same**	**Increase**	**p**
Fruit and vegetables	Intervention	9	0	32	0.0004
	Control	16	1	25	0.21
Breakfast cereal	Intervention	5	20	16	0.027
	Control	10	21	11	1.00
Potatoes pasta or rice	Intervention	13	12	16	0.71
	Control	8	15	19	0.052
Oil rich fish intake	Intervention	8	19	14	0.29
	Control	11	22	9	0.82
Sweet foods	Intervention	24	1	16	0.27
	Control	21	1	20	1.00
Chips	Intervention	19	17	5	0.0066
	Control	16	19	6	0.052
Savoury snacks	Intervention	17	16	8	0.11
	Control	19	12	10	0.14

**Table 7:** Comparison of changes in Eating Habits between Intervention and Control Groups at 24 weeks*

**Food Group**	**Odds Ratio**	**95% Confidence Interval**	**p**
Increased Fruit and Vegetables	2.11	(0.81, 5.52)	0.13
Increased Cereal intake	1.66	(0.50, 5.52)	0.40
Increased Potatoes, Pasta or Rice	0.77	(0.31, 1.92)	0.57
Increased Oil Rich Fish intake	1.78	(0.58, 5.45)	0.30
Decreased Sweet Foods intake	1.67	(0.60, 4.66)	0.32
Decreased Chips intake	1.58	(0.56, 4.44)	0.38
Decreased Crisps & Savoury Snacks	0.74	(0.30, 1.82)	0.51

*Analyses are logistic regression models adjusted for clustering by centres.

### Smoking cessation

Although the study was not powered to detect changes in smoking status these data were examined for completers and also using a sensitivity analysis. Both analyses showed a higher percentage of control participants still smoking at week 24 in comparison to intervention participants, however between group differences were not significant (Table 8).

**Table 8:** Comparison of Smoking Status between Intervention and Control Groups

	**Intervention**	**Control**	**Odds Ratio**	**95% Confidence Interval**	**p**
**Completers**					
Smoking at Week 6	9 (18.8%)	6 (11.3%)	1.74	0.40 to 7.66	0.46
Smoking at Week 24	17 (42.5%)	25 (58.1%)	0.43	0.14 to 1.34	0.14
**Sensitivity analysis**					
Smoking at Week 6	29 (42.6%)	23 (32.9%)	1.38	0.63 to 3.04	0.42
Smoking at Week 24	45 (66.2%)	52 (74.3%)	0.58	0.25 to 1.36	0.20

Analyses are logistic regression models adjusted for varenicline use and clustering by centres.

### Process evaluation

Nineteen intervention and 21 control participants were interviewed. Four emergent themes (group interactions, advisors interactions with groups and mechanisms relating to individual participants and specific to the dietary intervention), were identified each of which comprised several sub-themes (Table 9). At week 24 most intervention participants who were successful at stopping smoking and minimising weight gain cited these as helping both their quit attempts and weight management efforts, as did participants who continued to smoke and were unsuccessful at minimising weight gain.

**Table 9:** Process evaluation emergent themes with sub-themes and illustrative quotations

**Theme**	**Sub theme with illustrative quotations**
**1. Group Interaction**	**Accountability:***meeting other individuals I think has been a bit of a challenge as well you don’t want to let the rest of the group down. [Quitter (Q), ≤3% weight gain]*
	**Sharing experiences & ideas:***If you’re getting a craving for a cigarette ….. you come to a group and the more people that’s in a group the more opportunity you got for an answer. Person A might say, ’Oh I do this’, Person B say, ’I’ll do this,’, and then you’ve got a choice to try it. [Q, ≤3% weight gain]*
	**Encouragement:***I mean the fact that you’ve got other people there who support you, willing to talk to you and wie the same sort of feelings as yourself………it makes you realise you know that well if someone else can get through it, so can you. [Q, >3% weight gain]*
	**Lack of group support:***one of the things I liked about it was that - there was five of us I think and I was a bit disappointed that 3 of them stopped coming quite early on, but I think it was because they all went back to smoking cigarettes. [Q, >3% weight gain]*
**2. Advisors interaction with groups**	**Advisors guidance & encouragement:***still smoking and you’re a failure….there’s not that negativity at all, whereas it’s always, ‘Yea, you are but you can still do this.’ Everything’s dealt with in a very, very positive manner.….. it makes me quite happy to be open and honest about I’ve not been too good here. [NQ, ≤3% weight gain]*
**3. Mechanisms relating to individual participants**	**Participant motivation:***I think I took my mind off the goal which was to stop smoking and have a healthier lifestyle…. I can’t seem to get myself again focussed enough to get back on track, to think, ‘No, this is not where I want to be at this time’. [NQ, >3% weight gain]*
	**Barriers to weight management:***When I first stopped the smoking I couldn’t stop eating sweet things, it was dreadful. [NQ, <3% weight gain]*
**4. Mechanisms specific to the intervention**	**Influence of tools:***: It’s quite nice to see how many steps you’ve done each day and I think it’s been a bit of challenge. [Q, ≤3%]*
	**Goal setting:***It was a 12-mile walk…. I found it quite easy because I had been building maself up for it over a period of weeks. I do the gym three times a week. Ma sessions - I’ve upped them from half-an-hour to an hour. [Q, >3% weight gain]*

*Q = quitter, NQ = non-quitter*.

### Economic evaluation

The overall cost of delivering the intervention was estimated at £80 per person. As no between group difference in % weight gain at 24 weeks was observed the calculation of QALYs gained was not relevant. NICE cite £20,000 and £30,000 per QALY as indicative values of a health service becoming less cost-effective. We can therefore estimate that a service costing £80 per person would have to produce 0.0027 QALYs to 0.0040 QALYs because: £80/0.0027 = £30,000 and £80/0.004 = £20,000. There is no single accepted estimate of the change in QALYs from a change in weight so we used a range of values identified from the literature and the results are shown in Table 10. The figures in the table are ‘thresholds’ because they are the minimum levels of weight change for cost-effectiveness, given the programme cost, and relationship between weight and QALYs. This analysis suggested that the intervention would only need to produce a slightly lower weight gain than the control group to be judged cost effective.

**Table 10:** Weight change necessary to provide QALY gains

**Cost of intervention £80 per person**			**Minimum change in kg based on higher QALY/kg figures**
	**Men**	**Women**	
£30 k/QALY-	−4.4	−1.3	−0.4
- required gain 0.0027 QALYs			
£20 k/QALY -	−6.7	−1.9	−0.6
- required gain 0.004 QALYs			

## Discussion

In comparison to the control group the intervention was not successful at reducing weight gain. Those successful at stopping smoking gained 3.9 kg, which was more than the 2.5 kg women attempting smoking cessation are reportedly willing to tolerate [18] but less than the 5 kg at 6 months reported in other research [9]. Continued professional/therapist contact has been shown to improve weight management and smoking cessation outcomes [19,20]. Interviews with intervention participants highlighted that the support and encouragement they received from both their fellow group members and advisors had helped with both weight management and quit attempt. This continued contact may have reduced anxieties regarding weight gain and thus encouraged cessation maintenance despite weight gain.

The intervention focussed on encouraging participants, who were likely to experience increased appetite and food cravings to choose healthier, less energy dense foods which would minimise weight gain. While between-group differences in changes in reported eating habits were non-significant there was evidence of improved eating habits compared to baseline, in the intervention group. Fruit and vegetable consumption improved significantly suggesting that fruit was eaten in place of some higher energy snacks. Consumption of breakfast cereals also improved. Previous research has shown that weight gain is less in adults who eat breakfast [21,22]. Both these changes suggest that efforts were being made by participants to adopt healthier eating patterns.

Interview data shows that some viewed changes to diet and physical activity only necessary if they were successful at stopping smoking and therefore at risk of weight gain. Failure to give up smoking may have led some participants to abandon efforts to change their diet or physical activity level.

We have demonstrated that it is possible, after appropriate brief training, for those with no prior formal nutrition training to deliver a nutrition and lifestyle intervention. Training existing staff to deliver additional information to those attempting smoking did not incur excessive costs. The use of existing staff would allow diet and lifestyle aspects to be integrated into the existing smoking cessation programme and avoid the additional expense of employing specialists such as dietitians or nutritionists. Individuals attempting smoking cessation develop a rapport with the cessation advisor taking the group and this relationship may increase the likelihood of dietary advice being acknowledged and implemented. Whilst a dietitian may have more in-depth knowledge it is unlikely that at dietitian would be able to routinely attend all smoking cessation programme sessions. Utilisation of existing personnel was also important to allow the results to be sustainable and transferable for national application.

Uptake from cessation classes into this study was less than hoped for and this may reflect that for some smoking cessation was their sole objective and they were not considering, or could not consider other concomitant lifestyle changes. It is possible that those who chose to join the study were not entirely representative of all those attempting smoking cessation, but they were at least a sizeable proportion. Consideration must also be given to the extra time commitment required of study participants.

Whilst uptake to the study was lower than hoped for, attrition was lower than the anticipated 75%. Research in the field of weight management has suggested that lower attrition rates are representative of some form of success [23]. The lower attrition rate in the present study may thus indicate success in that for participants the programme fulfilled a role by providing additional support and assistance for them whilst attempting lifestyle changes.

The present study was not powered to investigate effects on cessation rates however the results suggest that smoking cessation was not compromised by the intervention. Comparison of the 6 week cessation rate for intervention participant with those of an earlier observation study carried out in Glasgow [24] showed those of the present study to be better (57% vs. 36%, p = 0.001). Improved cessation rates in the present study may in part reflect increased use of varenicline. Most study participants (59%) chose varenicline to aid their quit attempt, compared to only 13% of subjects in the study by Bauld and colleagues [24]. Cessation rates for both groups deteriorated between week 6 and 24 and it is reasonable to assume the same pattern is likely throughout Glasgow. However at week 24 the cessation rate in intervention participants was 34%, much better than expected and close to the week 4 cessation rate reported for the Glasgow wide service. This suggests that participation in the intervention arm of the study may have conferred some benefit in terms of maintenance of cessation. Improved cessation rates may not result directly from the dietary intervention per se but to other factors such as continued professional/therapist contact and reduced anxiety regarding weight gain. Interview data supports this. Intervention participants were conscious that exhaled CO levels would be checked and this provided motivation to refrain from smoking as a high reading, they felt, would be interpreted as failure and would let the group down. Remaining abstinent they felt also provided encouragement to the other group members.

Although between group differences in quit rates were not statistically significant in the present study the higher quit rate in intervention participants would be compatible with a real effect. Reverse power analysis using the present data showed that a study to prove an effect of this size would require 165 per group using completer data, and 539 per group using sensitivity analysis data.

### Economic evaluation

The figures provided (Table 10) are not precise calculations but are intended to be indicative of the relatively modest changes in weight that would be required given the level of costs. This may suggest that using a different protocol or providing the intervention to a different group may well be cost-effective. This supports the argument that the “non-significant” difference in the primary outcome measure should not end research in this area. The figures should be used with caution and as a starting point for debate only, for several reasons. First, they do not consider any cost savings from avoiding weight-related diseases. For example, if we included the chances of developing type 2 diabetes and included the lifetime costs of this illness the required weight reductions would fall. In addition, we have not considered that controlling weight may help people to stop smoking – any QALY gains from people helped to stop smoking have also not been included. There may also be benefits that have not been considered by this research, such as the spill-over effects of education in diet and exercise on the remainder of the family. Any benefits of this type would also help to make the programme cost-effective

### Limitations

Recruitment to research studies is likely to attract more motivated volunteers. The observation that weight gain in our control participants was similar to intervention participants may reflect their willingness or interest in making lifestyle changes in addition to smoking cessation. The qualitative data support this suggestion. The majority of control participants interviewed reported setting themselves diet and or physical activity goals and had sought help from other sources to achieve these.

The advisors who participated in the study and delivered the intervention also conducted the smoking cessation classes for control participants. Whilst aware of the need to avoid contamination of control classes it is possible that issues regarding weight and diet were answered in more depth, thus influencing the behaviour of control participants. It is not possible to quantify this potential contamination, but in retrospect, may have been avoided had advisors who had not been given additional training been recruited and allocated to control classes. However this would have been difficult in practice as randomisation of classes did not take place until week 3 and participants had already developed a rapport with the advisor running that class. Changing advisor at this point may have undermined attempts at smoking cessation.

Dietary practices were assessed using the Dietary Targets Monitor, a short food frequency questionnaire developed for use in the 1995 Scottish Health Survey. Formal comparison between this short questionnaire and a fuller and widely used food frequency questionnaire [14] showed that the dietary targets monitor had the capability to monitor intakes for changes towards national dietary targets for key foods and nutrients but did not very accurately predict results for some food groups when the study numbers were below 300. All methods of assessing dietary intake have limitations including mis-reporting [25]. For the present study it was important to identify a tool which was acceptable to the study population and not too onerous to complete, as this may have had adverse effects on retention rates. For these reasons and to gather as comprehensive a data set as possible the dietary targets monitor was used.

## Conclusions

Reductions in the prevalence of smoking and obesity are both public health priorities for healthcare services throughout the UK. The period following smoking cessation has been highlighted as a time when the risk of weight gain is increased [6]. The present study explored a way to tackle these two important health issues, and provides some evidence to guide future practice and research needs.

Not everyone attending smoking cessation programmes will be interested in additional lifestyle advice but the present study has shown that support can be made available for those who are interested. The present intervention was not successful at reducing weight gain. However improved quit rates in the intervention group suggest that continued contact with advisors may have reduced participants’ anxieties regarding weight gain and thus encouraged cessation maintenance despite weight gain. Provision of dietary advice may have assisted some in smoking cessation and improved health from this point of view, but also improved food choice. This could have additional benefits to health by furthering the reduction in risk of coronary heart disease. Improved diet composition may lead to better health even in those who failed to stop smoking. The cost of the intervention was relatively modest with only some reduction in post cessation weight gain required for the intervention to be judged cost-effective. It is important that research continues in this area as there is some evidence that the negative effects of obesity could outweigh the health benefits achieved through reductions in smoking prevalence [26,27].

## Competing interest

The authors declare there are no conflicts of interest.

## Authors’ contribution

MEJL & CRH conceived of the study and participated in its design. WSL participated in the study design, coordinated the study and drafted the manuscript. PK conducted and coded interviews and analysed all qualitative data. MM provided expertise in process evaluation and directed qualitative data analysis. HM carried out quantitative statistical analysis. AW carried out the economic evaluation. SB provided expertise in behavioural change training. All authors contributed to the writing of the manuscript and critically reviewed the final version submitted for publication. All authors read and approved the final manuscript.

## Funding source

The research was commissioned by the Food Standards Agency UK (FSA). The FSA had no role in the study design, collection, analysis or interpretation of data, or the decision to submit the paper for publication.

## Pre-publication history

The pre-publication history for this paper can be accessed here:

http://www.biomedcentral.com/1471-2458/12/389/prepub
